# Valorization of American Barrel-Shoot Wastes: Effect of Post Fermentative Addition and Readdition on Phenolic Composition and Chromatic Quality of Syrah Red Wines

**DOI:** 10.3390/molecules25040774

**Published:** 2020-02-11

**Authors:** Berta Baca-Bocanegra, Julio Nogales-Bueno, José Miguel Hernández-Hierro, Francisco José Heredia

**Affiliations:** Food Color and Quality Laboratory, Section of Nutrition and Food Science, Facultad de Farmacia, Universidad de Sevilla, 41012 Sevilla, Spain

**Keywords:** phenolic compounds, anthocyanins, wood, byproducts, color, wine

## Abstract

The influence of post fermentative addition of American barrel-shoot wastes on phenolic composition and chromatic quality of Syrah red wines has been evaluated as an environmentally sustainable alternative to the conventional winemaking for avoiding the common color loss of red wines elaborated in warm climates. American oak wood byproducts added were previously classified by hyperspectral image analysis according to the amount of phenolic compounds transferred to the extraction media. After that, wines were elaborated under different maceration conditions by applying only one proportion of wood (12 g L^−1^) and two different maceration procedures (simple and double addition) and were compared with a traditionally macerated Syrah red wine (CW, no wood addition). Results proved the effectiveness of the moderate postfermentative addition of oak wood byproducts to stabilize the color of wines and to provoke lower color modification along the time, producing color wines chromatically more stable for a better aging. In the case of double addition, the adsorption of the pigments during the maceration presents a stronger effect on the color than copigmentation and polymerization by cause of the copigments extracted from the wood.

## 1. Introduction

Color is one of the main characteristic defining the quality of wines and usually the first attribute perceived by the consumer, who want to get high quality wines. Phenolic compounds participate in several sensory attributes such as astringency, bitterness and color. Among them, anthocyanins extracted from grape skin are the principal compounds involved in the color of red wines and their interactions with other phenolic compounds (i.e., copigments), normally colorless, allow improving the color stabilization of aged wines by copigmentation reactions [1,2]. The amount of anthocyanins is influenced by a number of factors such as cultivar, growing region, climate, and growth conditions [3,4,5,6,7]. Under warm climatic conditions, it is a usual pattern the lack of copigmentation phenomena, which contribute to color stabilization, and it is hampered by the shortfall of pigments and copigments [1]. In this scenario, it is desirable to improve the extraction of phenolic compounds or to obtain additional polyphenols from other sources that contribute to assist in the copigmentation phenomena and hence in color stabilization. In these regards, different techniques such as alternative maceration procedures [8,9,10], electron beam irradiation [11] or grape processing by high hydrostatic pressure [12] have been developed to enhance the extraction of grape components responsible for the color, obtaining different results.

Wood from the manufacture of barrels is a natural source of phenols that, used in the initial stages of vinification in red wines, are expected to modify the conditions of the medium and affect their organoleptic characteristics, specifically, the color. The contribution of wood is expected to improve the stabilization of the wine color, contributing to the phenomena of copigmentation [13,14,15] or preventing the oxidation of colored compounds during winemaking maturation [16,17], especially when they are subjected to aging process. Although oak wood has been used for centuries for the elaboration of the barrels where the wine is stored during aging, the addition of cooperage byproducts such as oak wood has been recently aimed at implementing the production of high quality red wines in the last years. Food industry generates high amounts of solid waste/by-products, which represent a main disposal problem for the industry. The aforesaid by-products are very promising sources of value-added substances, with particular emphasis to the retrieval technologically important secondary metabolites. Over the next few years, food processing waste management might be rapidly expanded.

Taking into account these considerations, the aim of the present work was to evaluate the influence of post fermentative addition of American barrel-shoot wastes on phenolic composition and chromatic quality of Syrah red wines. This technique was applied as an oenological alternative to the conventional winemaking for avoiding the common color loss of red wines elaborated in warm climates.

In previous works carried out in our lab, near-infrared spectroscopy has been used for the screening of the extractable polyphenolic compounds in wood cooperage by-product demonstrating the suitability of this technique for predictive purposes [18,19]. Development of rapid and reliable models for the screening of extractable polyphenols will allow greater versatility and efficiency for the decision-making in the winemaking process on the adequacy and/or dosage of these by products according to the requirements of the wine. Taking into account the promising results of the developed methods and in order to optimize the procedure of this work, the added byproducts were previously classified by hyperspectral image analysis according to the amount of phenolic compounds transferred to the extraction media using the method described in Baca-Bocanegra, Nogales-Bueno, Hernandez-Hierro and Heredia [19].

## 2. Results and Discussion

### 2.1. Phenolic Compound Analysis

At the end of the maceration process (30 days for simple wine (SW) and 60 days for double wine (DW)) a significant increase (*p* < 0.05) of the content of total phenols was observed both in SW and in DW with respect to their respective control (Table 1). This increase was related to the transfer, during the maceration, of wood phenolic compounds such as phenolic acids and ellagitannins. As expected, DW had higher values than SW for this parameter since in this case a double amount of shavings was added during a longer period of maceration (12 g L^−1^ shavings for 30 days and a second addition of 12 g L^−1^). On the other hand, the contribution of wood to the content of flavan-3-ols was practically negligible by reason of their scarce presence in the wood. Regarding the content of anthocyanins, SW and DW experienced a fall in the level of pigments, very slight in the case of the simple and significant addition (*p* < 0.05) in the double addition treatment. This drop in anthocyanins content was consistent with the results previously obtained by other authors [2,20]. This effect may be related to polymeric anthocyanins which were created during the early steps of winemaking by condensation reactions between the anthocyanins themselves and other compounds given up by the wood or initially present in the wine as flavan-3-ols. In addition, the added wood could adsorb the anthocyanin compounds on its surface causing a decrease in its concentration especially when the amount of added wood was higher and the maceration period was longer [21].

During the stabilization phase (120 days from the start of the post-fermentative maceration), differences in the phenolic content between the two SW and DW wines tended to be reduced. Total phenols suffered a drop in both SW and DW with respect to CW although these differences were not significant. On the other hand, the falls experienced by the anthocyanins in SW and DW with respect to the control are significant (*p* < 0.05). This typically occurs as a result of reactions such as oxidation, polymerization and/or hydration [22].

The percentage of polymerization was 60.00, 66.81 and 64.16% for CW, SW and DW respectively. The higher percentage of polymerization in wood macerated wines indicated higher proportions of more stable anthocyanins and therefore more chromatic stability in these wines.

At the end of the maceration phase, there was an important difference in the fall experienced by flavan-3-ols and anthocyanins in DW with respect to SW. However, throughout the stabilization phase, there were no significant differences for these parameters between SW and DW, the levels being comparable and even slightly higher for the double addition test. This compensation may be related to the protective effect exerted by ellagitannins [23], added in greater quantity in the case of the double addition of shavings.

In the stabilization phase, there was also a significant decrease in the flavan-3-ols in SW and DW with respect to the control, probably as a consequence of their involvement in the reactions with anthocyanins [24,25].

### 2.2. Color Analysis

Table 2 shows the CIELAB color parameters (L *, a *, b *, C * _ab_, and h _ab_) for the different elaborated wines (CW, SW and DW) in three different stages of the winemaking process: addition of the wood (day 0), end of the maceration (day 30 for the simple addition test and day 60 for the double maceration test) and end of the stabilization process (day 120).

At the end of the maceration phase, the largest color extraction corresponded to the control wines, which showed the lowest values of lightness and hue (L * and h _ab_) and the highest chroma values (C * _ab_), which it translates into darker wines, with more chromatic intensity and red-blue hues. These results were consistent with the higher content of anthocyanins in the control wines during the maceration step, especially in the double addition test. However, during the stabilization stage, these results were inverted and it is the SW and DW wines that presented larger chromatics and smaller hues than the control, that is, wines with greater chromatic intensity and more bluish red hues than the control, although these differences they were only significant in case of hue. There were no differences between the tests and the control with respect to lightness. The double test had a higher hue, ie, redder orange hues, which were in agreement with a larger amount of free anthocyanins, less stable and less percentage of polymerization than the simple addition test.

**Figure 1:**
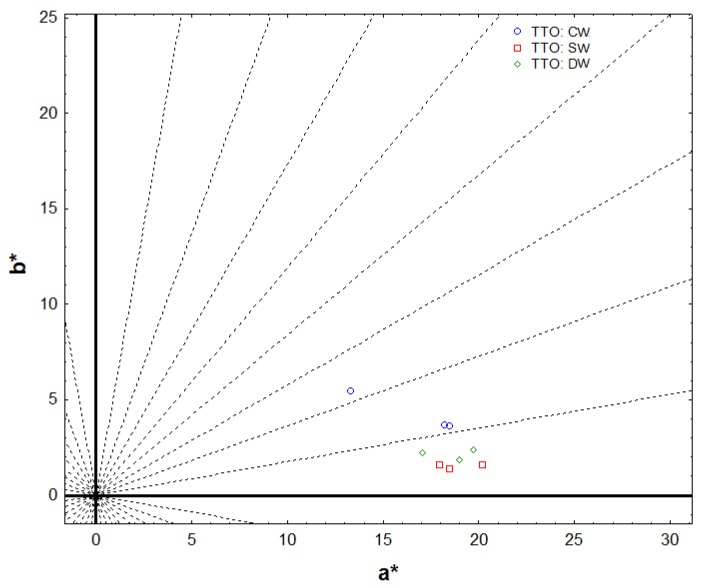
Location of wines at the end of the stabilization phase (120 days) on the CIELAB (a * b *)-diagram.

This fact can be observed in the color diagram (Figure 1). The different position of the traditionally produced wines and the wines made with the addition of raw wood, at the end of the stabilization phase, make it possible to objectively establish their chromatic characteristics. All the wines are located in the first quadrant (positive values of a * and b *). However, SW and DW wines have smaller b values that result in wines with higher purple or reddish-blue hues than control wines.

In order to evaluate the color stability of each wine, the CIELAB color differences (ΔE * _ab_) and the differences for the different color parameters (ΔL *, ΔC * _ab_ and Δh _ab_) were calculated for each wine (CW, SW and DW) considering the end point of the stabilization stage with respect to the initial point (day 0, shaving addition) (Table 3).

The addition of raw wood caused a positive effect not only on color density (chroma) and hue but also on color stability. This means that, during the stabilization stage, the loss of color experienced by the wines made by adding wood is less than in the control wine. As expected, this loss of color was less in the simple addition test than in the double test. The color differences were mainly due to qualitative differences (Δh_ab_) since it was the only parameter for which there were significant differences between the treatments and the control; CW, SW and DW experienced positive variations of the hue towards redder-orange tones, these differences being smaller in the case of SW. All wines experienced losses in the chroma but the differences between them are negligible. With regard to lightness, this parameter increased in the three wines, with SW experiencing a smaller increase although the differences between them were not significant.

In order to evaluate the influence of the post-fermentation addition at the end of the stabilization stage, the color differences between SW and DW with respect to CW (SW_120_ vs. CW_120_ and DW_120_ vs. CW _120_) were determined (Table 4). The greatest color difference was seen in the case of the simple addition (4.51 vs. 3.76 units) although these differences between the tests were not significant. Taking into account that ΔE*_ab_ greater than 3 CIELAB units indicate appreciable differences by the human eye [26], the color differences caused by the post-fermentative addition of raw wood both simple and re-addition or double are appreciable for the human eye.

## 3. Material and Methods

### 3.1. Oak Wood Byproducts

American non-toasted oak wood (*Quercus alba* L.) shavings, generated by sawing of the staves during the process of making barrels, were used for this study (Tonelería Salas S.L., Bollullos Par del Condado, Huelva, Spain). Upon receipt, wood samples were screened using 2 mm and 10 mm mesh sieves placed in tandem. Only the shaves which were retained between them were used in the assay.

In order to consider wood phenolic heterogeneity described in bibliography [27,28,29,30], sieved oak wood shavings were divided into different subsamples and their extractable phenolic content were predicted using the hyperespectral method described in Baca-Bocanegra, Nogales-Bueno, Hernandez-Hierro and Heredia [19]. In the previously mentioned work, a quantitative model was developed by modified partial least squares (MPLS) regression to predict extractable phenolic content of wood from the spectral information. For it, spectra of a calibration set samples was used as the independent variables and their wood extractable total phenolic content as dependent variables. The robustness of the model was tested using validation set samples, which did not belong to the calibration set, as external validation. The method developed in the preceding work was used in the present study to predict the extractable content of total phenols in all wood samples. All samples present a Mahalanobis distance (H) < 3 in the space used to develop the model and then all samples are within the applicability of the obtained model and could be predicted.

Oak wood shavings was dosed in the different tanks, taking into account the predicted extractable phenolic content, in such a way that this parameter does not suppose a new factor of variability in the vinification assay.

### 3.2. Winemaking

Red wines were elaborated under different maceration conditions by applying only one proportion of wood (12 g L^−1^) and two different maceration procedures and were compared with a traditionally macerated Syrah red wine.

Wines were made from grapes *Vitis vinifera* L. var. Syrah grown in a vineyard belonging to “Condado de Huelva” Designation of Origin (DO), in the southwest of Spain (37°22′01′′N, 06°32′29′′W). The “Condado de Huelva” Designation of Origin is a typical warm climate region where the average temperature for the last 10 years, from 21 June to 21 September, was around 25 °C, although it ranged between 14 and 41 °C (data provided by Instituto de Investigación y Formación Agraria y Pesquera (IFAPA), Junta de Andalucía, Spain).

The vinification process was carried out as previously described in Rivero et al. [20]. Briefly, about 900 kg of grapes were harvested in 2016 vintage at optimum technological maturity and in good sanitary conditions. The destemmed and crushed grapes were allocated in six stainless steel tanks of 220 L capacity to run the alcoholic fermentation by adding 25 g hL^−1^ of selected *Saccharomyces cerevisiae* yeast (Viniferm BY, Agrovin, Ciudad Real, Spain). To improve skin maceration, the mash was manually punched down once a day (6 days). After this, to remove the solid parts the mash was drawn off, and the free run wines were racked to nine 50 L stainless steel tanks.

**Figure 2:**
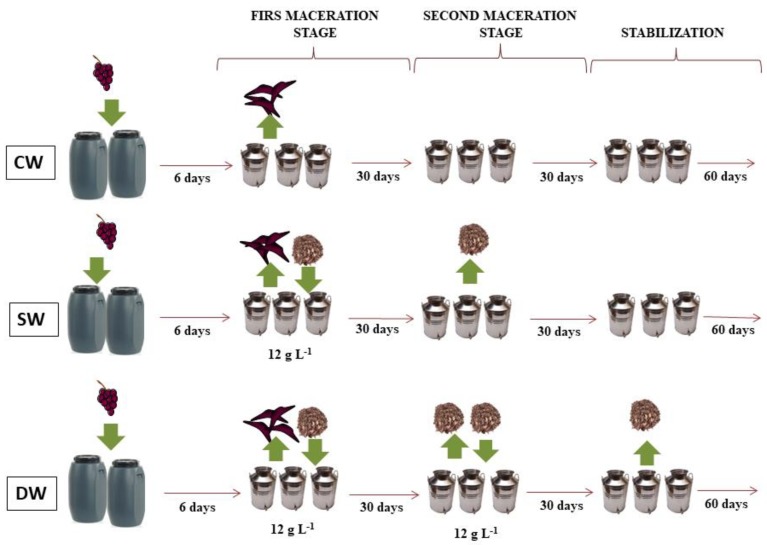
Schematic representation of the wine elaboration process.

The first procedure involved the oak wood maceration for 30 days (SW), in a second strand, oak wood was readded and macerated for 30 additional days (DW). A control wine was also elaborated (CW). Synchronously to the wood addition (without wood in control wines), selected *Oenococcus oeni* lactic acid bacteria (VINIFERM Oe 104, 14 mL hL^−1^, Agrovin, Ciudad Real, Spain) were inoculated to run the malolactic fermentation. At the end of the aforesaid procedure, sulfur dioxide levels were adjusted (free sulfur dioxide circa 100 mg L^−1^ in all wines). The wines were preserved in the stainless steel tanks during 120 days until the end of the stabilization step. A schematic workflow is provided in Figure 2. Three samples were taken into account in this study: initial point: 0 days, addition of wood, wood removal: after 30 days for SW and 60 days for DW and end of stabilization point (120 days). As can be noted, the end of the stabilization process is the same for both procedures.

### 3.3. Phenolic Compound Analysis

Total phenolic content was determined using the Folin–Ciocalteu method [31]. Gallic acid was used as a standard for construction of the calibration curve and the concentration of total phenols was expressed as gallic acid equivalent.

Flavan-3-ols content was determined following a modification of Vivas et al. [32] using the DMACA (4-dimethylaminocinnamaldehyde) as reagent. A calibration curve of (+)–catechin was used for quantification and results were expressed as (+)–catechin equivalent.

Both Folin–Ciocalteu and DMACA analyses were performed on an Agilent 8453 UV–visible spectrophotometer (Palo Alto, USA), equipped with diode array detection (DAD).

Anthocyanin content was determined by chromatographic analysis following a modification of the method of García-Marino et al. [33] as described elsewhere in Hernández-Hierro et al. [34]. Results were expressed as mg of malvidin-3-*O*-glucoside equivalents.

All determinations were performed in triplicate.

### 3.4. Color Analysis

The visible spectra (380–770 nm) was measured in triplicate at constant intervals (Δλ = 2 nm) with an Agilent 8453 UV–Vis spectrophotometer (Palo Alto, USA), using 2 mm path length glass cells and distilled water as white reference. CIE 1964 10° standard observer and the CIE *D65* illuminant were used as references to calculate the tristimulus values recommended by the Comission Internationale de l′Éclairage [35]. The CIELAB space was used and parameters measured included: Lightness (*L* *), red-green coordinate (*a* *, −*a* *), yellow-blue coordinate (*b* *, −*b* *). From a* and b* coordinates another two color parameters are defined: the hue angle (*h _ab_)* and chroma (C * _ab_) which indicate qualitative and quantitative aspects of color respectively. Calculations were made using the CromaLab^®^ software (Sevilla, Spain) [36].

### 3.5. Statistical analysis

Univariate analyses of variance (ANOVA) and Tukey post *hoc test* were applied to look for differences in the obtained mean values for each parameter. The statistically significant level was considered at α = 0.05. All statistical analyses were performed using Statistica v.8.0 software (StatSoft Inc., OK, USA, 2007).

## 4. Conclusions

The addition of oak wood shavings to red wine elaboration could represent an oenological alternative to the conventional winemaking for wines with unbalanced copigment/pigment proportion that is the case of winemaking in warm climates conditions. This addition, thanks to the transfer of wood compounds, improves the copigment proportion, avoiding the anthocyanin degradation (by copigmentation and polymerization processes), and leads to more suitable wines for the aging process.

In this work, simple post-fermentative addition of oak wood led to better results. This process resulted in positive effects on wine color, giving wines with more bluish hues than the wines traditionally produced. Moreover, results proved the efficiency of the post-fermentative maceration with oak wood, mainly the simple addition, to stabilize the color of wines and to provoke lower color modifications along the time, producing wines chromatically more stable for a better aging. In the case of double addition, the adsorption of the pigments during the maceration presents a stronger effect on the color than copigmentation and polymerization (color stabilization) by cause of the copigments extracted from the wood.

Finally, the use of cooperage by-products as a source of copigments for wine leads to a sustainable and competitive cooperage industry, through waste reduction and by-product valorization.

## Figures and Tables

**Table 1:** Polyphenols content (mg L^−1^) for the different elaborated wines (CW, SW and DW) in three different stages of the winemaking process. For each compound, different letters in the same row indicate statistical differences (Tukey test, α = 0.05).

	CW30	SW30	CW60	DW60	CW120	SW120	DW120
	Mean ± SD	Mean ± SD	Mean ± SD	Mean ± SD	Mean ± SD	Mean ± SD	Mean ± SD
Total Phenols	2019.19a ± 36.07	2466.32b ± 218.38	2174.45a ± 157.95	2503.72b ± 119.89	2304.90a ± 292.92	1984.79a ± 221.36	2052.87a ± 165.94
Total flavan-3-ols	141.21a ± 7.24	136.55a ± 8.24	129.11a ± 9.28	102.24b ± 9.31	137.66a ± 16.91	70.43b ± 4.17	88.03b ± 8.87
Total Anthocyanins	150.20a ± 11.12	141.17a ± 4.84	132.44a ± 19.04	68.74b ± 3.49	113.57a ± 40.77	15.77b ± 1.10	23.63b ± 6.70
Total non acylated anthocyanins	67.83a ± 2.00	66.02a ± 3.62	62.99a ± 7.05	35.67b ± 2.15	54.66a ± 18.63	6.85b ± 0.56	11.33b ± 3.50
Total acetyls anthocyanins	46.53a ± 2.34	45.27a ± 0.61	43.50a ± 5.47	24.14b ± 1.28	36.94a ± 12.60	6.70b ± 0.39	9.77b ± 2.44
Total coumaroyls anthocyanins	36.51a ± 7.28	30.55a ± 1.16	26.63a ± 6.64	9.60b ± 0.21	22.64a ± 9.55	2.89b ± 0.35	3.21b ± 0.77
Total acylated anthocyanins	82.71a ± 9.20	75.49a ± 1.40	69.79a ± 12.10	33.41b ± 1.34	59.25a ± 22.14	9.25b ± 0.55	12.64b ± 3.22
Delphinidin-3-*O*-glucoside	2.90a ± 0.19	2.91a ± 0.13	2.81a ± 0.36	1.61b ± 0.07	2.53a ± 0.68	0.60b ± 0.04	0.78b ± 0.13
Petunidin-3-*O*-glucoside	5.56a ± 0.15	5.39a ± 0.16	5.20a ± 0.65	3.00b ± 0.12	4.63a ± 1.46	1.00b ± 0.04	1.38b ± 0.28
Peonidin-3-*O*-glucoside	5.53a ± 0.88	4.62a ± 0.96	4.50a ± 0.61	2.35b ± 0.10	3.46a ± 1.11	0.52b ± 0.06	0.66b ± 0.13
Malvidin-3 *O*-glucoside	54.85a ± 1.14	54.12a ± 2.43	51.49a ± 5.92	29.72b ± 1.86	45.05a ± 15.38	5.74b ± 0.45	9.53b ± 2.96
Delphinidin-3-*O*-(6′acetyl)-glucoside	1.56a ± 0.70	1.48a ± 0.72	1.60a ± 0.19	1.18b ± 0.07	1.42a ± 0.30	0.78b ± 0.02	0.83b ± 0.06
Petunidin-3-*O*-(6′acetyl)-glucoside	2.18a ± 0.25	2.08a ± 0.06	1.84a ± 0.30	1.13b± 0.04	1.74a ± 0.53	0.72b ± 0.02	0.79b ± 0.09
Peonidin-3-*O*-(6′acetyl)-glucoside	1.03a ± 0.19	1.06a ± 0.09	1.04a ± 0.25	0.44b ± 0.03	0.83a ± 0.28	0.64b ± 0.02	0.60b ± 0.03
Malvidin-3-*O*-(6′acetyl)-glucoside	4.42a ± 0.13	4.61a ± 0.56	4.44a ± 0.31	2.50b ± 0.20	3.65a ± 1.05	0.83b ± 0.04	1.09b ± 0.22
Delphinidin-3-*O*-(6′-*p*-coumaroyl)glucoside (*trans*)	38.70a ± 2.63	37.39a ± 0.93	35.93a ± 4.87	20.24b ± 1.03	30.65a ± 10.54	5.08b ± 0.32	7.80b ± 2.13
Malvidin- 3-*O*-(6′ caffeoyl)-glucoside (trans)	1.42a ± 0.23	1.11a ± 0.08	0.77a ± 0.39	0.50a ± 0.07	0.70a ± 0.23	0.62a ± 0.02	0.59a ± 0.02
Cyanidin-3-*O*-(6′-*p*-coumaroyl)glucoside	1.48a ± 0.16	1.36a ± 0.17	0.95a ± 0.37	0.66a ± 0.18	0.98a ± 0.26	0.37b ± 0.01	0.39b ± 0.02
Petunidin-3-*O*-(6′-*p*-coumaroyl)glucoside (trans)	3.48a ± 1.28	2.66a ± 0.25	2.07a ± 0.67	0.88b ± 0.22	1.80a ± 0.60	0.41b ± 0.01	0.39b ± 0.01
Malvidin-3-*O*-(6′-*p*-coumaroyl)glucoside (cis)	2.91a ± 1.88	1.21a ± 0.51	0.96a ± 0.19	0.45b ± 0.04	0.66a ± 0.12	0.65a ± 0.05	0.61a ± 0.07
Peonidin-3-*O*-(6′-*p*-coumaroyl)glucoside (trans)	2.86a ± 0.05	2.90a ± 0.70	2.98a ± 1.30	1.32b ± 0.09	2.44a ± 0.60	0.56 b± 0.09	0.56b ± 0.07
Malvidin-3-*O*-(6′-*p*-coumaroyl)glucoside (trans)	26.05a ± 3.93	23.00a ± 0.41	20.59a ± 3.78	7.47b ± 0.59	17.77a ± 8.29	1.88b ± 0.23	2.37b ± 0.70

**Table 2:** CIELAB color parameters (L *, a *, b *, C *_ab_, and h _ab_) for the different elaborated wines (CW, SW and DW) in three different stages of the winemaking process. For each parameter, different letters in the same row indicate statistical differences (Tukey test, α = 0.05).

Stage		CW	SW	DW
		Mean ± SD	Mean ± SD	Mean ± SD
**Initial** **(0 day)**	L *	74.34 ± 0.53	74.83 ± 0.87	73.90 ± 0.76
	a *	27.18 ± 0.80	28.88 ± 0.88	28.51 ± 0.55
	b *	−0.99 ± 0.25	−1.72 ± 0.25	−1.68 ± 0.14
	C * _ab_	27.20 ± 0.79	28.93 ± 0.89	28.23 ± 0.54
	h _ab_	−2.10 ± 0.57	−3.40 ± 0.43	−3.53 ± 0.37
**Maceration** **(30 days)**	L *	79.17a ± 0.54	83.44b ± 0.22	
	a *	19.18a ±0.72	15.19b ± 0.17	
	b *	2.41a ±0.31	3.39b ± 0.18	
	C * _ab_	20.22a ± 0.61	15.57b ± 0.13	
	h _ab_	6.29a± 1.36	12.57b ± 0.77	
**Maceration** **(60 days)**	L *	79.96a ± 1.24		81.31a ± 0.45
	a *	20.25a ± 0.89		19.49a ± 0.25
	b *	1.00a ± 0.15		1.55b ± 0.06
	C * _ab_	20.77a± 0.68		19.55a ± 0.26
	h _ab_	2.74a ± 0.54		4.54b ± 1.14
**Stabilization** **(120 days)**	L *	80.81a ± 0.52	80.54a ± 1.76	80.92a ± 1.33
	a *	18.01a ± 0.63	18.89a ± 1.17	18.60a ± 1.35
	b *	3.91a ± 0.45	1.50b ± 0.10	2.14b ± 0.26
	C * _ab_	17.87a ± 1.32	18.95a ± 1.17	18.73a ± 1.35
	h _ab_	10.94a ± 0.71	4.55b ± 0.37	6.59c ± 0.92

**Table 3:** Color differences for each wine (CW, SW and DW) considering the end point of the stabilization with respect to the initial point (day 0, wood addition). For each parameter, different letters in the same row indicate statistical differences (Tukey test, α = 0.05).

		CW	SW	DW
Stage		Mean ± SD	Mean ± SD	Mean ± SD
**Stabilization** **(120 days)**	ΔE *_ab_	13.50a ± 3.20	12.00a ± 2.65	12.76a ± 1.90
	ΔL *	6.47a ± 0.58	5.71a ± 2.24	7.02a ± 1.48
	ΔC _ab_	−9.99a ± 3.21	−9.99a ± 1.90	−9.51a ± 1.38
	Δh _ab_	13.04a ± 1.24	7.94b ± 0.75	10.12b ± 1.28

**Table 4:** Color differences between SW and DW with respect to CW (SW_120_ vs. CW_120_ and DW_120_ vs. CW_120_) at the end of the stabilization stage. For each parameter, different letters in the same row indicate statistical differences (Tukey test, α = 0.05).

		Post-Maceration Treatment	
		SW	DW
Stage		Mean ± SD	Mean ± SD
**Global**	ΔE * _ab_	4.51a ± 2.69	3.76a ± 2.56
	ΔL *	−0.36a ± 2.08	0.10a ± 1.14
	ΔC _ab_	2.12a ± 2.88	1.52a ± 3.16
	Δh _ab_	−6.33a ± 0.51	−4.34b ± 0.61
